# Long noncoding RNA SNHG4 promotes renal cell carcinoma tumorigenesis and invasion by acting as ceRNA to sponge miR-204-5p and upregulate RUNX2

**DOI:** 10.1186/s12935-020-01606-z

**Published:** 2020-10-19

**Authors:** Jie Wu, Tingting Liu, Lulu Sun, Shaojin Zhang, Gang Dong

**Affiliations:** 1 Department of Ultrasound Intervention, The First Affiliated Hospital, Zhengzhou University, 1 Jianshe Dong Road, Zhengzhou, 450052 Henan China; 2 Department of Urology Surgery, The First Affiliated Hospital, Zhengzhou University, Zhengzhou, Henan China

**Keywords:** Renal cell carcinoma, SNHG4, miR-204-5p, RUNX2, ceRNA

## Abstract

**Background:**

Long noncoding RNAs (lncRNAs) are involved in the tumorigenesis and progression of human cancers, including renal cell carcinoma (RCC). Small nucleolar RNA host gene 4 (SNHG4) is reported to play an essential role in tumor growth and progression. However, the molecular mechanisms and function of SNHG4 in RCC remain undocumented.

**Methods:**

Quantitative real-time polymerase chain reaction (qRT-PCR) was performed to examine expression levels of SNHG4 in RCC tissue samples and cell lines. Cell counting kit-8, western blotting, activities of caspase-3, -8, and -9, wound-healing, and transwell invasion assays were performed to explore cell proliferation, apoptosis, migration, and invasion. The interaction among SNHG4, miR-204-5p, and RUNX2 was verified by bioinformatic analysis, a luciferase gene report, qRT-PCR, western blot analysis, and RNA immunoprecipitation assays. Xenograft mouse models were carried out to examine the role of SNHG4 in RCC in vivo.

**Results:**

SNHG4 was highly expressed in RCC tissue samples and cell lines, and its upregulation was significantly involved in node involvement, distant metastasis, and reduced overall and relapse-free survival of patients with RCC. SNHG4 acted as an oncogenic lncRNA with promoted RCC cell proliferation, migration, invasion, and inhibited apoptosis. SNHG4 boosted tumor growth in xenograft mouse models. Mechanistically, SNHG4 functioned as a competing endogenous RNA (ceRNA) for sponging miR-204-5p, leading to the upregulation of its target RUNX2 to promote RCC cell proliferation and invasion.

**Conclusion:**

SNHG4 and miR-204-5p might be indicated in RCC progression via RUNX2, suggesting the potential use of SNHG4/miR-204-5p/RUNX2 axis in RCC treatment.

## Background

It is estimated that about 73,820 new cases and 14,770 deaths from kidney malignancy occur in 2019 [1]. Clear cell renal cell carcinoma (ccRCC) is the most common histological subtype of kidney malignancy, accounting more than 80% of cases [2]. Currently, standard surgical radical nephrectomy or partial nephrectomy is the most optimal therapeutic option; however, nearly 25% of ccRCC cases will undergo local relapse or distant metastasis after tumor resection [3]. Thus, timely diagnosis and treatment will be vital for the improvement of patients’ prognosis [4]. Therefore, it is essential to explore the further mechanisms of RCC progression, which will lead to a better understanding of tumor biology and develop more effective target therapies for RCC [5].

Long noncoding RNAs (lncRNAs) are classified according to their non-protein-coding potentials with lengths larger than 200 nucleotides [6]. In the past decades, accumulating evidence showed that lncRNAs exert an important effect on the processes of transcriptional regulation, post-transcriptional regulation, and epigenetic modification [7]. The dysregulation of lncRNAs has been well documented in the tumorigenesis and development of human malignancies [8, 9]. For example, enhanced lncRNA ARSR expression was shown in RCC‐initiating cells, related with dismal survival, and contributed to the sunitinib resistance in RCC patients [10]. Results from Zeng et al. showed that lncRNA 00312 could prohibit RCC cell proliferation and migration and boost apoptosis in vitro by repressing miR-34a-5p and enhancing expression of ASS1 [11]. Some lncRNAs named small nucleolar RNA host genes (SNHGs), which can encode small nucleolar RNAs (snoRNAs) [12, 13]. Recent studies have found that SNHGs can play specific predictive roles for the prognosis of some types of cancer, including cancers of the colorectum, pancreas, and breast [14–16]. Among these SNHGs, aberrant expression of SNHG4, located in 5q31.2, has been reported in various human malignancies, such as prostate cancer, osteosarcoma, cervical cancer, and hepatocellular carcinoma [17–21]. For example, lncRNA SNHG4 facilitates cervical tumorigenesis and cancer progression by sponging miR-148a-3p and upregulating the expression of c-Met [18]. SNHG4 boosts osteosarcoma advancement by competitively binding miR-224-3p [20]. To date, the expression pattern, clinical effects, and the underlying molecular mechanism of SNHG4 in RCC are still poorly understood.

In the present study, we aimed to check the expression levels of SNHG4 in RCC tissue samples and cell lines, and explore the biological behaviors of SNHG4 in RCC carcinogenesis by in vitro and in vivo experiments. Subsequently, we also determined the underlying molecular mechanisms of SNHG4 in RCC progression. Our investigation may provide potential biomarkers for RCC targeted treatment.

## Materials and methods

### Study subjects

Ninety-nine RCC tumor tissue (TT) samples and matched adjacent nontumor tissue (ANT) samples were available from cases who underwent radical nephrectomy at the First Affiliated Hospital of Zhengzhou University between January 2011 and October 2011. All patients were ultimately diagnosed with RCC according to histopathological evaluation. They previously did not receive adjuvant anti-cancer therapy before surgery. All collected tissue samples were immediately snap-frozen in liquid nitrogen and stored at − 80 °C until required. The study was performed complying with the ethical standards of the Helsinki Declaration, and ethical approval was received from the ethics committee of this hospital. Written informed consent was also obtained from all the patients. Detailed information was shown in Additional file 1: Table S1.

### Cell lines and cell culture

Five human RCC cell lines (Caki-1, Caki-2, ACHN, 786-O, and 769-P) were cultured in standard Dulbecco’s modified Eagle’s medium (DMEM, Corning) supplemented with 10% fetal bovine serum (FBS; Gibco) and 2 mM l-glutamine (Corning), 100 units/ml penicillin and 100 μg/ml streptomycin. Human proximal tubular epithelial cells (HK-2) were cultured in keratinocyte serum-free medium supplemented with 5% FBS. All these cells were obtained from American Type Culture Collection and grown in a humidified incubator with 5% CO_2_ at 37 °C.

### Cell transfection

Small interfering RNAs (siRNAs) for SNHG4 and Runt-related transcription factor 2 (RUNX2) and control siRNA were provided by Invitrogen (Clontech Laboratories, Inc.). The full-length complementary cDNA of human SNHG4 was synthesized and cloned into the expression vector pcDNA3.1. Vectors containing short hairpin RNAs (shRNA) targeting SNHG4 (shSNHG4), full-length SNHG4, and negative controls, provided by GeneChem Co., were packed into lentivirus [Lv] vector. MiR-204-5p mimics, inhibitor, and negative controls [NCs] were provided by RiboBio. The sequence was confirmed by DNA sequencing. The above RNA oligoribonucleotides or constructs were transfected into RCC cell lines using lipofectamine 2000 (Invitrogen) according to the manufacturer’s instructions.

### Quantitative reverse transcription–PCR (qRT-PCR)

Total RNA was extracted from RCC tissues and whole cell lysate using TRIzol (Invitrogen) according to the manufacturer’s protocol. Using a Reverse Transcription Kit (Takara), cDNA was converted from RNA and was performed for further qRT -PCR with SYBR Green (Applied Biosystems) according to the manufacturer’s protocol. Expression was normalized to endogenous controls, GAPDH, U6, and calculated using relative quantification method [22]. The primers used in the qRT-PCR were shown in Additional file 1: Table S2.

### Western blotting analysis

RIPA buffer (Thermo Scientific) was used to isolate the proteins in RCC tissue specimens and cell lines. Equivalent proteins (30 μg) were run on SDS-PAGE and transferred onto polyvinylidene fluoride (PVDF) membranes. Then, we blocked the membranes with 5% BSA for 1.5 h and then incubated with primary antibodies: GAPDH (1:1000, Abcam), RUNX2 (1:1000, Abcam), PCNA (1:1000, Abcam), and Bcl-2 (1:1000, Abcam) overnight at 4 °C. And then, membranes were incubated with secondary antibody. The protein bands were finally detected using enhanced chemiluminescence (Pierce, Rockford, IL, USA). Protein expression levels were quantitated by normalization against GAPDH with ImageJ version 1.36 (https://imagej.nih.gov/ij/).

### Luciferase reporter assay

We utilized online software starBase 3.0 to predict the potential binding sites of SNHG4 and miR-204a-5p or miR-204a-5p and RUNX2 3′ untranslated region (UTR). These synthetic fragments, obtained via PCR amplification, were cloned into the pGL3-Basic Vector (Promega, Madison, WI, USA) to construct a wide-type dual-luciferase reporter plasmid (SNHG4 wt). A Mutagenesis Kit (QIAGEN, California, USA) was utilized to produce a mutant-type (mut) SNHG4 vector (SNHG4 mut). 769-P and ACHN cells were transfected with luciferase vector reporter plasmid and negative control. After 24 h, the cells were collected and tested with a luciferase assay kit (Promega) according to the supplier’s protocol.

### RNA immunoprecipitation (RIP) assay

RIP assay was performed by an EZMagna RIP™ RNA Binding Protein Immunoprecipitation Kit (Millipore) following the supplier’s recommendations. In short, 769-P and ACHN cells were lysed with a RIP lysis buffer, and the cell extract was incubated with magnetic beads conjugated with specific Ago2 antibody (Abcam) or control IgG (Millipore) at 4 °C for 6 h. Finally, the purified RNAs were tested by qRT‐PCR.

### Subcellular location

After nuclear and cytoplasmic fractions were separated using the PARIS Kit (Thermo Fisher Scientific, Waltham, Mass., USA), the nuclear and cytoplasmic RNA was isolated and then were tested by qRT-PCR. GAPDH and U1 were utilized as an internal control for cytoplasmic and nuclear RNA expression, respectively.

### Cell counting kit-8 (CCK-8) assay

RCC cell proliferation was detected using CCK-8 kit (Beyotime Institute of Biotechnology, Shanghai, China) following the manufacturer’s recommendations. 769-P and ACHN cells (1.0 × 10^3^) were cultured in a 96-well plate for 24 h, and then transiently transfected with siRNAs or overexpression plasmids. Approximately 10 μl of CCK8 reagent was added per well and then cultured at 37 °C for 2 h. The absorbance at 575 nm in each well was measured at 1, 2, 3, and 4 h by a microplate reader (Thermo Fisher Scientific).

### Caspases activity

The transfected 769-P and ACHN cells in different groups were seeded in 6-well plates. Cells were cultured for 24 h at 37 °C, 5% CO_2_. Then, cells were collected and lysed in 50 μl of ice-cold cell lysis buffer. The activity of caspase was detected using caspase-3, -8, and -9 colorimetric assay kit, provided by Abcam. Samples were read at OD405 nm in a microtiter plate reader (Benchmark, Bio-Rad, USA).

### Cell migration and invasion assay

Cell migration and invasion assays were performed using Transwell chambers (8.0 μm pore size, BD Biosciences, USA) without (for migration) or with matrigel (for invasion). Cells were seeded in the top compartment of the transwell, and the bottom chambers were filled with 500 μl medium containing 10% FBS. After staining with crystal violet for 15 min, the migrated or invaded cells were counted under a light microscope (Olympus).

The scratch assay was conducted to assess cell migration capability. RCC cells were grown to 100% confluence in 24-well plates. A sterile 200 μl pipette tip was utilized to produce a clear line in the wells. The initial gap breadth (0 h) and the residual gap breadth (24 h) were taken using a light microscope (Nikon, Tochigi, Japan). Experiments were repeated at least three times.

### Animal experiments

ACHN cells were stably infected with the lentivirus plasmids containing shSNHG4, shNC, pcDNA3.1/SNHG4, or pcDNA3.1 vectors, respectively. For tumor formation assay, ACHN cells (5 × 10^6^) were implanted subcutaneously into a single side of male athymic BALB/c nude mice (4–5 weeks old, n = 5 in each group). All mice were kept under specific pathogen-free conditions. Tumor growth of mice was monitored every 5 days, and mice were killed after 5 weeks. Tumor volume was calculated using the formula, 0.5ab^2^ (a, longitudinal diameter; b, latitudinal diameter). This study was carried out according to the Guide for the Care and Use of Laboratory Animals of the NIH (Bethesda, MD). The Committee of the Ethics of Animal Experiments of Zhengzhou University approved the protocol.

### Immunohistochemistry (IHC)

After deparaffinized and rehydrated, sections were subjected to high-pressure for antigen retrieval. Hydrogen peroxide was utilized to inactivate endogenous peroxidase activity. BSA was used to block nonspecific binding. Sections were incubated with rabbit polyclonal anti-Ki-67 (1: 800. Abcam) overnight at 4C. IHC staining was visualized using the DAKO REAL EnVision Inspection System (DAKO).

### Bioinformatics and statistical analysis

Data mining analyses were conducted following the Lnclocator [23], StarBase 3.0 (http://starbase.sysu.edu.cn/), and UALCAN plotter (http://ualcan.path. uab.edu/index.html) databases. Data are expressed as the mean ± SD. Statistical analyses were conducted using SPSS 19.0 (SPSS Inc., Chicago, IL, USA) and GraphPad Prism version 8.0 (GraphPad Inc., La Jolla, CA, USA). One-factor analysis of variance (ANOVA) test, student’s *t* test, spearman correlation test, and chi‐squared test were performed when appropriate. The Kaplan–Meier method and the log-rank test were performed to calculate overall survival (OS) and relapse-free survival (RFS) curves. Cox’s proportional hazards regression model was used to analyze the independent prognostic factors. *P*-value < 0.05 was considered to be statistically significant.

## Results

### Association of SNHG4 expression with clinical features and prognosis in patients with RCC

**Fig. 1:**
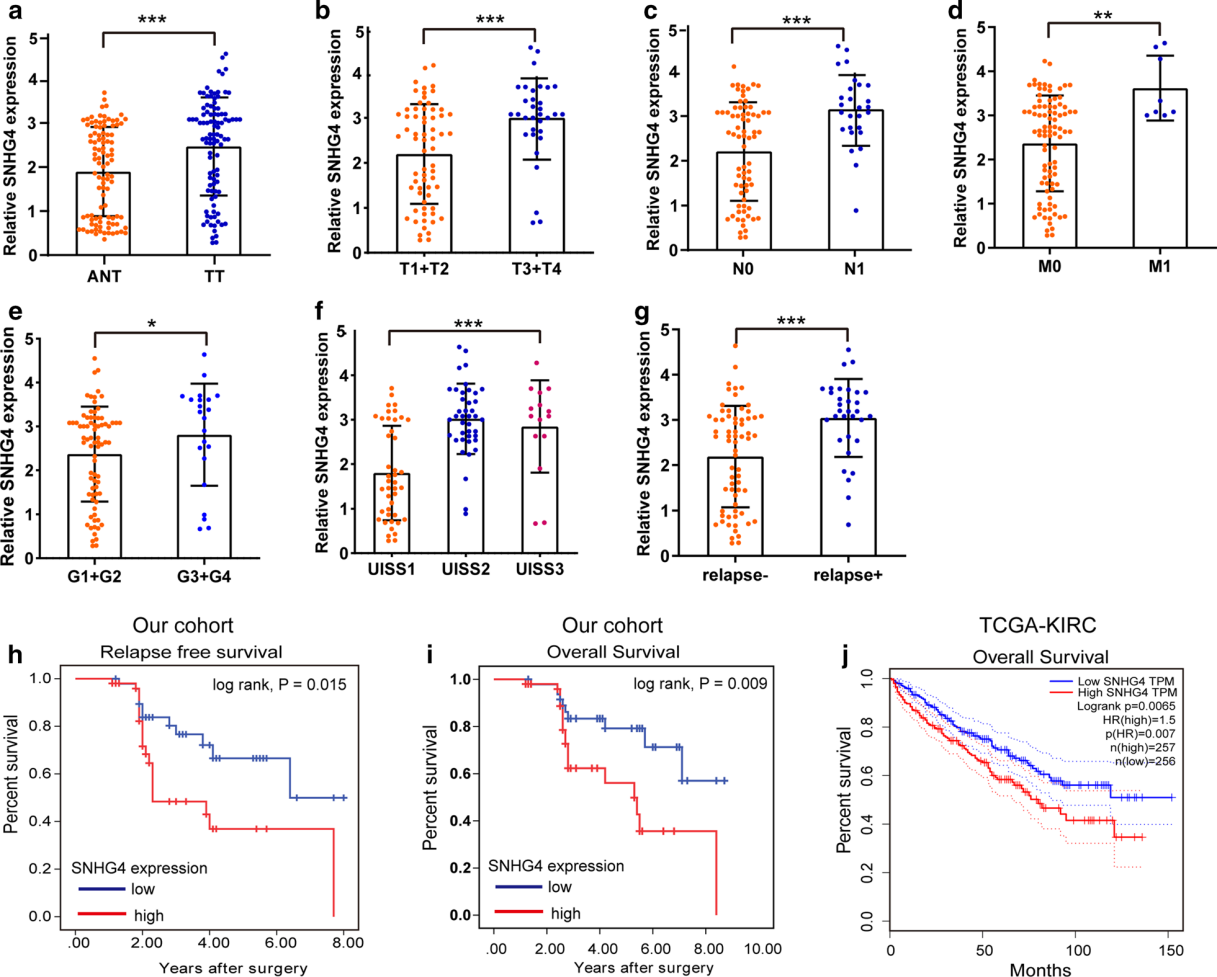
SNHG4 expression in human RCC tissue samples, and its association with prognosis. **a** qRT-PCR assay analysis of SNHG4 expression in TT and ANT human renal tissues in our cohort (n = 99). **b**–**g** qRT-PCR assay showing that high expression of SNHG4 was significantly associated with aggressive T stage (**b**), node invasion (**c**), metastasis status (**d**), poor tumor grade (**e**), UISS score category (**f**), and relapse status (**g**). Data were obtained using the 2^−ΔΔCT^ method and were normalized to GAPDH levels. **h**–**j** Kaplan–Meier survival curves according to high and low SNHG4 expression in patients with RCC from the Zhengzhou cohort (**h**, **i**) and TCGA-KIRC data set (**j**). Tumor classification and stage were referred to the 7th edition of UICC (2009) on cancer staging system. RCC: renal cell carcinoma; TT: tumor tissues; ANT: adjacent non-cancerous tissues; UISS: University of California, Los Angeles, Integrated Staging System; TCGA: the cancer genome atlas; KIRC: kidney renal clear cell carcinoma. *P < 0.05; **P < 0.01; ***P < 0.001

To explore the effects of SNHG4 on RCC aggression, we used qRT-PCR assay to determine the expression levels of SNHG4 in the TT and matched ANT samples from our cohort containing 99 RCC patients. The expression of SNHG4 in TT was significantly higher than that in ANT (Fig. 1a), which was also confirmed in the dataset from the cancer genomic atlas (TCGA)-kidney renal clear cell carcinoma (KIRC) (Additional file 1: Fig. S1A). Furthermore, high expression of SNHG4 was significantly associated with advanced T stage, node invasion, distant metastasis status, high University of California, Los Angeles, Integrated Staging System (UISS) score category, poor tumor grade, and relapse status (Fig. 1b–g and Additional file 1: Fig. S1B-D). Kaplan–Meier survival curves showed that high expression of SNHG4 was related to dismal RFS and OS (Fig. 1h, i) in patients with RCC from the Zhengzhou cohort and TCGA-KIRC dataset (Fig. 1j). The multivariate cox survival analyses suggested that high SNHG4 expression was an independent factor of poor RFS (hazard ratio [HR] = 1.77, 95% confidence interval [CI] 1.04–3.01) and OS (HR = 2.48, 95% CI 1.02–6.03; Table 1). Furthermore, qRT-PCR assay showed the expression levels of SNHG4 in five RCC cell lines were significantly upregulated compared with HK-2, a renal epithelial cell line (Additional file 1: Fig. S1E). These results unambiguously demonstrated that SNHG4 might act as an oncogenic lncRNA in RCC.

**Table 1:** Univariate and multivariate analyses of factors related to survival in patients with RCC

Parameters	Univariate analysis hazard ratio (95% CI)		P value	Multivariate analysis, hazard ratio (95% CI)*		P value
*Relapse free survival*						
T stage						
T3 + T4/T1 + T2	2.97 (1.38–6.39)		0.005	1.31 (0.45–3.68)		0.607
N stage						
N1/N0	1.74 (0.79–3.83)		0.170	1.32 (0.82–2.11)		0.248
M stage						
M1/M0	1.85 (0.55–6.16)		0.319			
Grade						
3 + 4/1 + 2	2.55 (1.41–4.62)		0.002	1.46 (1.05–2.04)		0.026
UISS category						
High + moderate/low risk	2.46 (1.44–4.21)		0.001	2.31 (1.11–4.83)		0.026
SNHG4						
High/low	2.33 (1.13–4.83)		0.022	1.77 (1.04–3.01)		0.035
*Overall survival*						
T stage						
T3 + T4/T1 + T2	2.42 (1.43–4.10)		0.001	1.53 (1.03–2.28)		0.037
N stage						
N1/N0	2.50 (1.09–5.72)		0.030	1.63 (0.88–3.03)		0.122
M stage						
M1/M0	1.65 (0.38–7.13)		0.499			
Grade						
3 + 4/1 + 2	2.26 (1.39–3.67)		< 0.001	2.12 (0.73–6.22)		0.170
UISS category						
High + moderate/low risk	2.17 (1.37–3.44)		0.001	1.95 (1.07–3.55)		0.029
SNHG4 expression						
High/low	3.24 (1.44–7.30)		0.005	2.48 (1.02–6.03)		0.045

UISS: UCLA Integrated Staging System

*Each patient was staged according to 2010 AJCC TNM classification [18]

### Overexpression of SNHG4 promotes cell proliferation, migration, invasion and inhibits apoptosis in RCC cell lines

**Fig. 2:**
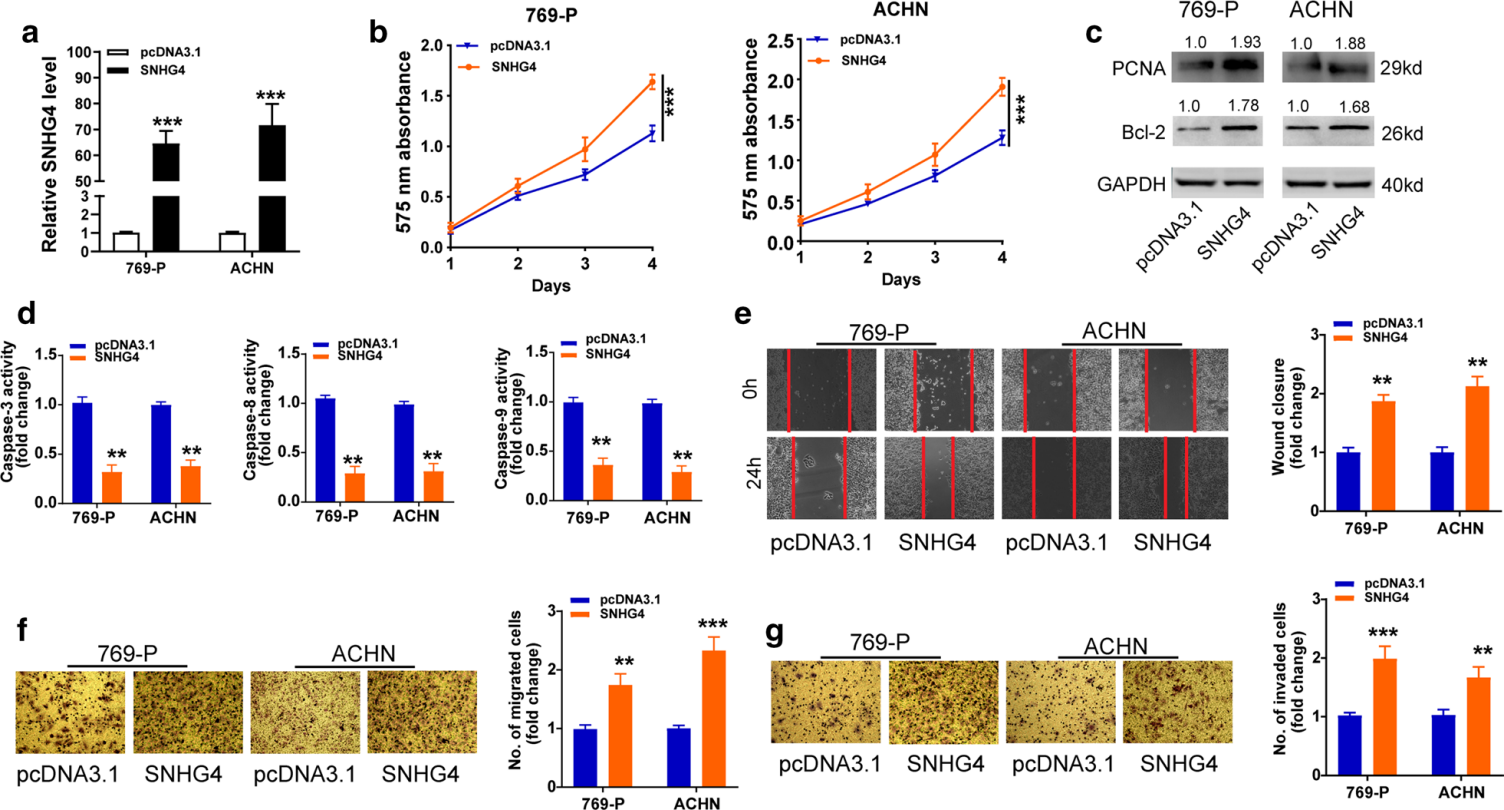
Overexpression of SNHG4 promotes cell proliferation, migration, invasion and inhibits apoptosis of RCC cells in vitro. **a** qRT-PCR assay analysis of the expression of SNHG4 in 769-P and ACHN cell lines transfection with pcDNA3.1/SNHG4 or pcDNA3.1. **b**, **c** CCK-8 assay (**b**), and Western blot assay (**c**) analysis of the proliferative ability of 769-P and ACHN cells transfected with pcDNA3.1/SNHG4 or pcDNA3.1. **d** The activity of caspase-3, -8, and -9 assay analysis of cell apoptosis of RCC cell lines after transfection with the indicated vectors. **e** The wound healing assay analysis of cell migration in 769-P and ACHN cell lines after transfection with the indicated vectors. **f, g** The transwell assay without (**f**) or with (**g**) matrigel analysis of cell migration and invasion in 769-P and ACHN cell lines after transfection with the indicated vectors. Data are presented as mean ± standard deviation from triplicate experiments. A *t*-test was used to evaluate the statistical significance as compared to the control. RCC, renal cell carcinoma. *P < 0.05; **P < 0.01; ***P < 0.001

To further explore the effects of SNHG4 on the progression of RCC cell lines, we transfected SNHG4 overexpression vector (pcDNA3.1/SNHG4) and control (pcDNA3.1) intro 769-P and ACHN cell lines. After transfection for 48 h, SNHG4 level was significantly elevated in the two RCC cell lines, as evidenced by qRT‐PCR analysis (Fig. 2a). Afterward, CCK-8 assays showed that cell viability was enhanced by transfection with SNHG4 overexpression vector in 769-P and ACHN cell lines compared with the pcDNA3.1 group. Western blot analysis showed that SNHG4 overexpression led to the enhanced expression levels of PCNA and Bcl-2 protein in 769-P and ACHN cell lines (Fig. 2c). Further, the activity of caspase-3, -8, and -9 assay found that SNHG4 overexpression significantly prohibited RCC cell apoptosis (Fig. 2d). The migratory and invasive ability was increased after cells transfected with SNHG4, as determined by wound healing and transwell assays (Fig. 2e–g). These results suggested that overexpression of SNHG4 could boost RCC cell proliferation and invasion, but inhibit cell apoptosis.

### Silencing of SNHG4 prohibits cell proliferation, migration, invasion, but enhances cell apoptosis in RCC cell lines

**Fig. 3:**
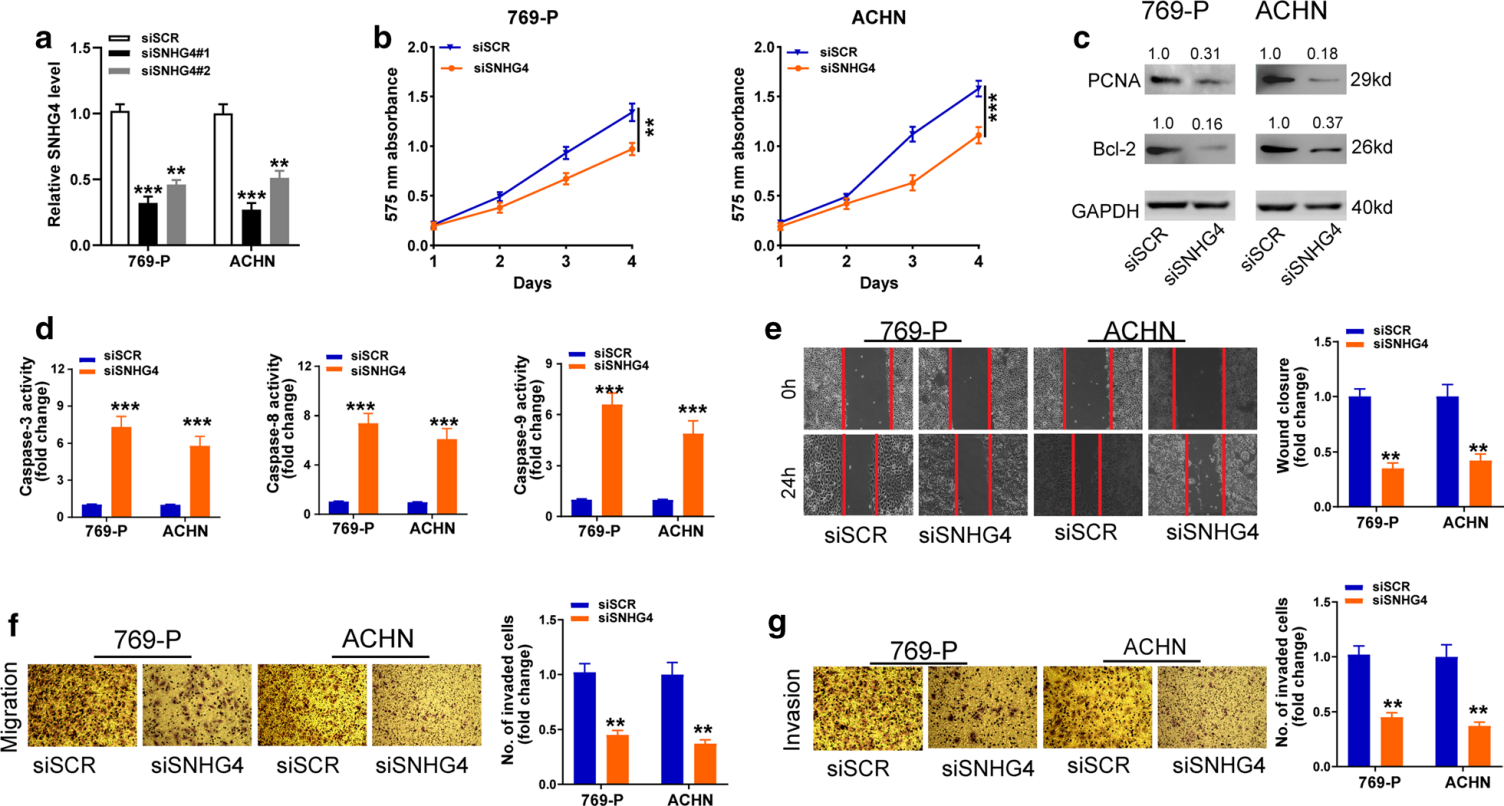
Knockdown of SNHG4 inhibits cell proliferation, migration, invasion, but promotes cell apoptosis in RCC cell lines. **a** qRT-PCR assay analysis of the level of SNHG4 in 769-P and ACHN cell lines after transfection with two parallel siSNHG4 (siSNHG4#1 and siSNHG4#2) or siSCR. **b**, **c** CCK-8 assay (**b**) and Western blot assay (**c**) analysis of the proliferative ability of 769-P and ACHN cells transfected with siSNHG4#1 or siSCR. **d** The activity of caspase-3, -8, and -9 assay analysis of cell apoptosis of RCC cell lines after transfection with the indicated vectors. **e** The wound healing assay analysis of cell migration in 769-P and ACHN cell lines after transfection with the indicated vectors. **f**, **g** The transwell assay without (**f**) or with (**g**) matrigel analysis of cell migration and invasion in 769-P and ACHN cell lines after transfection with the indicated vectors. Data are presented as mean ± standard deviation from triplicate experiments. A *t*-test was used to evaluate the statistical significance as compared to the control. SCR: scramble; RCC: renal cell carcinoma. *P < 0.05; **P < 0.01; ***P < 0.001

To determine whether silencing of SNHG4 could affect the biologic behaviors of RCC cells, we transfected two parallel siRNAs targeting the coding region of SNHG4 (siSNHG4#1 and siSNHG4#2) into 769-P and ACHN cell lines, respectively. qRT-PCR analysis showed that compared with the scramble (siSCR) control, siSNHG4#1 had higher knockdown efficiency (Fig. 3a). So, siSNHG4#1 was used to perform the following knockdown experiments. The CCK-8 assay showed that the proliferation of 769-P and ACHN cell lines was attenuated by knockdown of SNHG4 (Fig. 3b). Moreover, the silencing of SNHG4 resulted in decreased PCNA and Bcl-2 protein (Fig. 3c). Additionally, we found markedly elevated activity of caspase-3, -8, and -9 in RCC cells transfected siSNHG4 compared with cells transfected siSCR (Fig. 3d). The migratory and invasive ability was markedly decreased after silencing of SNHG4 expression in 769-P and ACHN cell lines, as evidenced by wound healing and transwell assays (Fig. 3e–g). These results suggested that SNHG4 knockdown could impede RCC cell growth and invasion.

### SNHG4 acts as a sponge of miR-204-5p in RCC cells

**Fig. 4:**
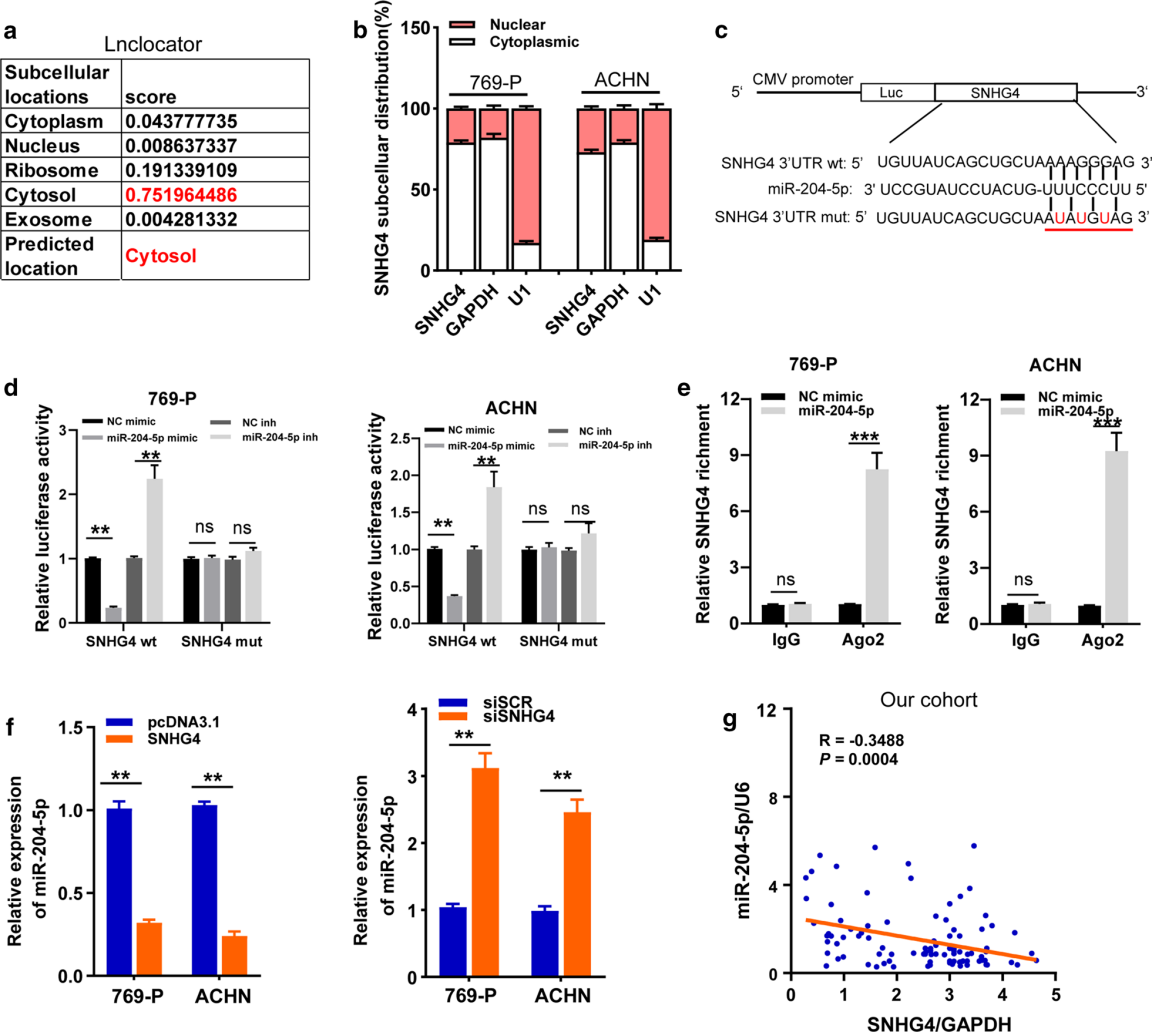
SNHG4 acts as a sponge of miR-204-5p in RCC cell lines. **a** Predicted subcellular localization of SNHG4 in RCC cells using the “lnclocator” algorithm. **b** qRT-PCR assay analysis of the subcellular localization of SNHG4 in RCC cells. GAPDH and U1 served as a cytoplasmic and nuclear localization marker, respectively. **c** The predicted binding sites of miR-204-5p with SNHG4 wt or mut were showed. **d** Luciferase activity assay analysis of 769-P and ACHN cell lines co-transfected with miR-204-5p mimic or inhibitor with SNHG4 wt or mut reporter gene. **e** Anti-Ago2 RIP assay analysis of 769-P and ACHN cell lines with transiently miR-204-5p mimic and control. The expression levels of endogenous SNHG4 were detected using qRT-PCR. **f** qRT‐PCR assay analysis of the expression of miR-204-5p in 769-P and ACHN cell lines after transfection with the indicated vectors. **g** Spearman’s correlation analysis of the correlation between SNHG4 and miR-204-5p in RCC tissues from our cohort. Data are presented as mean ± standard deviation from triplicate experiments. A *t*-test was used to evaluate the statistical significance as compared to the control. SCR: scramble; RCC: renal cell carcinoma; ns, not significant. *P < 0.05; **P < 0.01; ***P < 0.001

**Fig. 5:**
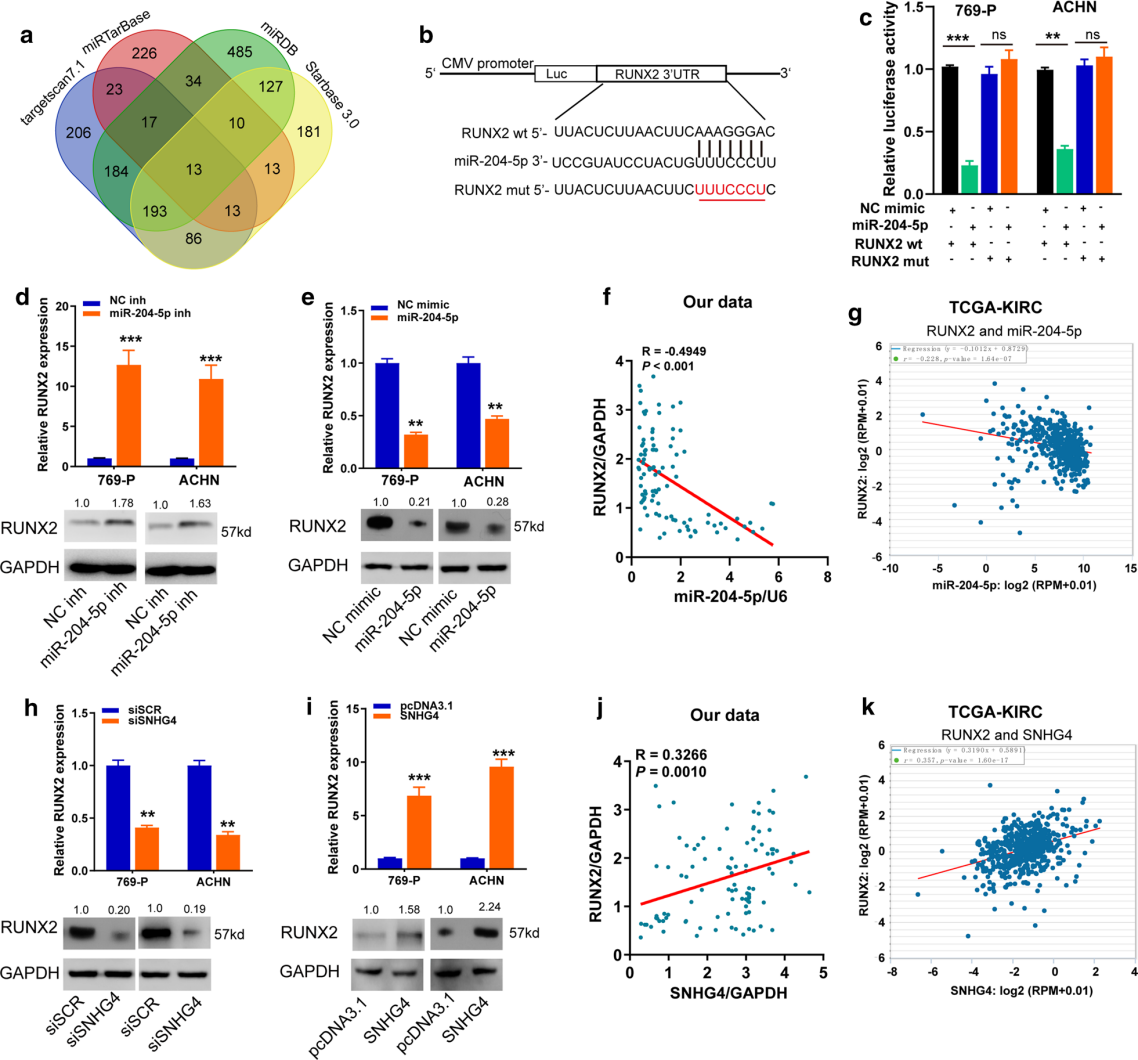
SNHG4 decoys miR-204-5p to upregulate its target gene RUNX2 in RCC cells. **a** A Venn diagram showed the number of genes identified as potential targets of miR-204-5p. **b** The miR-204-5p putative binding sequences and corresponding mutant sites of RUNX2 3′UTR. **c** Dual-luciferase reporter assay analysis of the luciferase activity in 769-P and ACHN cell lines after co-transfection with miR-204-5p mimic and construct containing wt and mut 3′-UTR of RUNX2. **d**, **e** qRT-PCR and western blot assay analysis of the expression levels of RUNX2 mRNA and protein in 769-P and ACHN cell lines after transfection with corresponding vectors. **f**, **g** Significantly inverse correlations between expression levels of miR-204-5p and RUNX2 mRNA were observed in human RCC tissues from the Zhengzhou cohort (**f**) and TCGA data set (**g**). **h**, **i** qRT-PCR and western blot assays analyses of the expression levels of RUNX2 mRNA and protein in 769-P and ACHN cell lines after transfection with corresponding vectors. **j**, **k** A positive correlation between SNHG4 and RUNX2 expression in human RCC tissues from our cohort (**j**) and TCGA (**k**) dataset. Data are presented as mean ± standard deviation from triplicate experiments. SCR: scramble; RCC: renal cell carcinoma; wt: wild-type; mut: mutant-type; ns: not significant. *P < 0.05; **P < 0.01; ***P < 0.001

A plethora of researches have confirmed that lncRNAs functioned as regulators through competitively sponging miRNAs, particularly when the lncRNAs mainly localized in cytoplasm [24, 25]. Indeed, results from the lnclocator and the further cellular fractionation experiments indicated that SNHG4 was preferentially localized to the cytoplasm of RCC cell lines (Fig. 4 A-B). Based on the starBase 3.0 online software, we found 40 miRNAs that might bind to SNHG4 (Additional file 1: Fig. S2A). Furthermore, the dataset from TCGA-KIRC showed that miR-204-5p expression possessed a negative correlation with SNHG4 and was significantly decreased in tumor tissues compared with normal renal tissues (Additional file 1: Fig. S2B, C). MiR-204-5p was also lowly expressed in the RCC cell lines than the normal renal cell lines (Additional file 1: Fig. S2D). So, we selected miR-204-5p for further investigation. The transfection efficiency of miR-204-5p mimic and inhibitor was validated by qRT-PCR analysis (Additional file 1: Fig. S2E). The binding sites of miR-204-5p with wt or mut SNHG4 were indicated in Fig. 4c. Next, using dual-luciferase reporter assays, we showed that miR-204-5p mimic significantly decreased, while miR-204-5p inhibitor increased the luciferase activity of SNHG4 wt reporter gene, but not the SNHG4 mut vector (Fig. 4d, e). Moreover, anti-Ago2 RIP assay in 769-P and ACHN cells with transiently overexpressing miR-204-5p showed that the expression levels of endogenous SNHG4 were specifically enriched in the miR-204-5p mimic group, compared with the NC group (Fig. 4e). Furthermore, overexpression of SNHG4 significantly inhibited, while knockdown of SNHG4 enhanced the expression of miR-204-5p in 769-P and ACHN cells (Fig. 4f). In addition, a significant negative correlation between SNHG4 and miR-204-5p in RCC tissues from our cohort (R = − 0.3488, *P* = 0.0004; Fig. 5g). These data clarified that SNHG4 could sponge miR‐204-5p in RCC cells.

### SNHG4 decoys miR-204-5p to upregulate its target gene RUNX2 in RCC cells

To find out genes sharing the regulatory role of miR-204-5p with SNHG4, we first predicted putative targets of miR-204-5p based on 4 independent algorithmic programs (targetscan7.1, miRDB, miRTarbase, starbase3.0), and 13 common targets were identified (Fig. 5a). Among these, RUNX2 was selected because it was a transcriptional factor which was upregulated and associated with TNM stage and poor survival according to the dataset from TCGA-KIRC (Additional file 1: Fig. 3a–e). To determine if RUNX2 is an authentic target of miR-204-5p, we utilized luciferase reporter assays. We demonstrated that the up-regulation of miR-204-5p significantly repressed the luciferase activity of RUNX2 wt 3′-UTR reporter plasmid, whereas no evident inhibition was observed in cells transfected with the mutant reporter plasmid (Fig. 5b, c). Moreover, silencing of miR-204-5p dramatically enhanced, while overexpression of miR-204-5p repressed the mRNA and protein expression levels of RUNX2 in 769-P and ACHN cells (Fig. 5d, e). Besides, Spearman’s correlation analysis showed a significant inverse relationship between RUNX2 mRNA and miR-204-5p expression in human RCC tissues from our cohort and TCGA-KIRC dataset (Fig. 5f, g). Further investigations showed that the upregulation and downregulation of SNHG4 positively affected RUNX2 expression at both the mRNA and protein levels in RCC cell lines (Fig. 5h, i). We also detected a positive correlation between SNHG4 and RUNX2 expression in human RCC tissues from our cohort and TCGA-KIRC dataset (Fig. 5j, k). Our data indicated the existence of a SNHG4/miR-204-5p/RUNX2 regulatory axis in RCC cells.

### MiR-204-5p reverses the promoting effect of SNHG4 on the proliferation and invasion of RCC cells

**Fig. 6:**
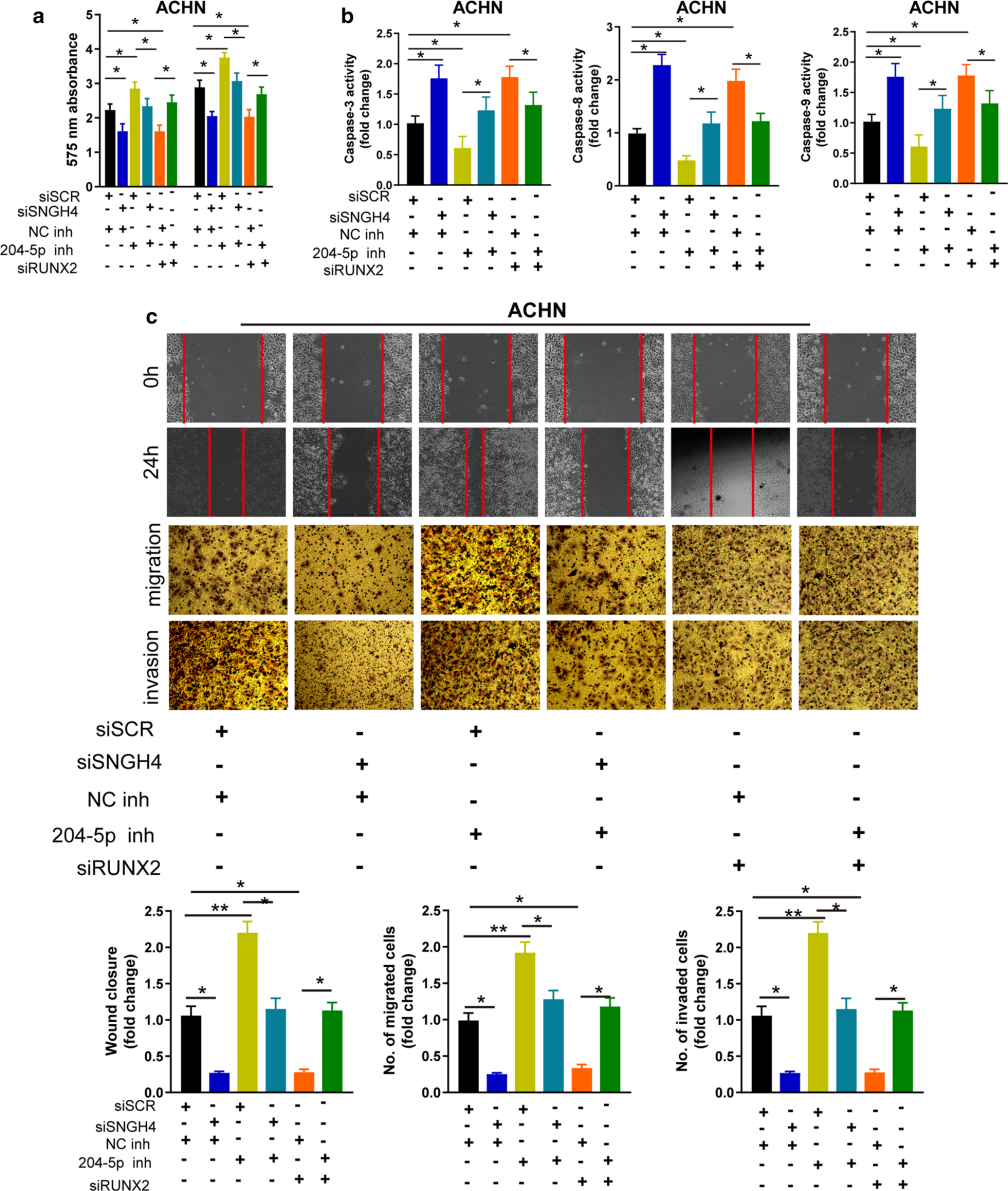
MiR-204-5p reverses the promoting effect of SNHG4 on the proliferation and invasion of RCC cells. **a** The CCK-8 assay analysis of the proliferative ability of ACHN cells after transfection the indicated vectors. **b** The activity of caspase-3, 8, and 9 assay analysis of cell apoptosis of ACHN cells after transfection with the indicated vectors. **c** Wound healing and transwell with or without matrigel assays analysis of cell migration and invasion in ACHN cells after transfection with the indicated vectors. Data are presented as mean ± standard deviation from triplicate experiments. SCR: scramble; RCC: renal cell carcinoma. *P < 0.05; **P < 0.01; ***P < 0.001

To investigate whether SNHG4 exerted its function via miR-204-5p/RUNX2 in RCC cells, we conducted rescue experiments. As shown in Fig. 6a–c, knockdown of the endogenous SNHG4 or RUNX2 markedly inhibited the proliferation, migration, and invasion, but promoted apoptosis of ACHN cells. Knocking down miR-204-5p alone dramatically enhanced the proliferative, migration, and invasion, but repressed apoptosis of ACHN cells. Moreover, silencing of miR-204-5p blocked the effects induced by SNHG4 or RUNX2 depletion. These data suggested that miR-204-5p reversed the enhanced roles of SNHG4 in the proliferative, migratory, and invasive capacity of RCC cells.

### SNHG4 promotes tumor growth of RCC in vivo

**Fig. 7:**
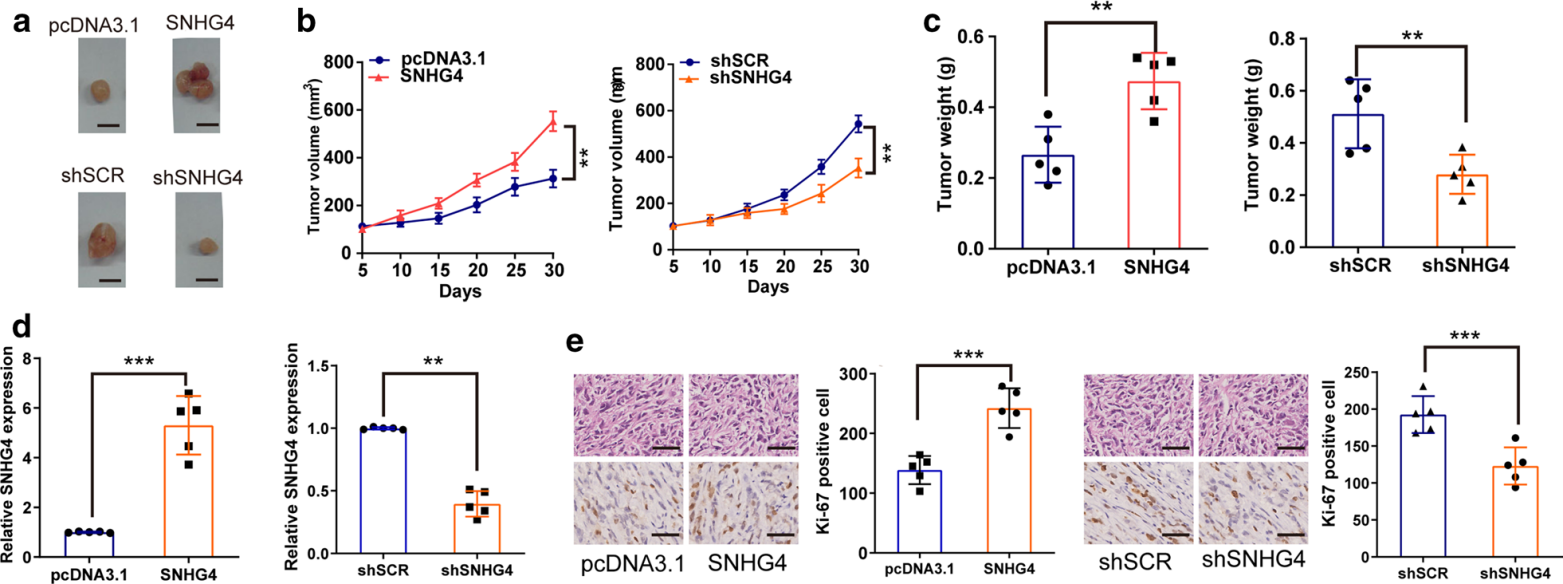
SNHG4 promotes tumor growth of RCC in vivo. **a**, **b** Measurement of the volumes of xenograft tumors. Scale bar, 1.0 cm. **c** Measurement of the weights of xenograft tumors. **d** qRT-PCR assay analysis of SNHG4 expression in xenograft tumors. **e** Ki-67 staining in xenograft tumor. Scale bar, 200 μm. Data are shown as the mean ± SD of three replicates. **P < 0.01; ***P < 0.001

To evaluate the effects of SNHG4 on in vivo tumor growth, we infected ACHN cells with stably overexpression or silencing Lv vectors and corresponding negative controls, and then implanted them subcutaneously into the mice, respectively (Fig. 7a). Compared with the NC group, the volumes and weight of tumors were remarkably enhanced in the SNHG4 overexpression groups, whereas were significantly repressed in the SNHG4 silencing groups (Fig. 7b, c). Furthermore, qRT-PCR assay verified the increased levels of SNHG4 expression in the overexpression groups, but significantly decreased levels in the SNHG4 silencing groups, compared with control tumors (Fig. 7d). Likely, the expression levels of Ki-67 staining by IHC assay were increased in tumors from SNHG4 overexpression groups but were decreased in tumors from SNHG4 silencing groups, compared with tumors from the corresponding control mice (Fig. 7e). These data suggested that SNHG4 contributes to RCC tumorigenesis in vivo.

## Discussion

In recent decades, many studies have found the critical function of lncRNAs in many types of human cancer, including RCC [11, 26, 27]. This evidence is of benefit to clarify the mechanistic basis of RCC. Herein, we explored the relationships between SNHG4 and clinical significance of RCC patients using the TCGA dataset and our cohort, and found that overexpressed SNHG4 was observed in RCC tissue and was associated with poor survival. We further found that SNHG4 promoted RCC cell proliferation, migration, invasion, and growth, but inhibited cell apoptosis in vitro and in vivo. Further mechanical studies demonstrated that SNHG4 functioned as a ceRNA to sponge miR-204-5p, then upregulate RUNX2, to promote RCC tumor progression. Our experiments suggested that SNHG4 plays an oncogenic role in the modulation of the properties of RCC cells.

Up to date, only a small proportion of lncRNAs and their biological functions have been elucidated. LncRNA SNHGs reside in the introns of host genes, which encode snoRNA and was recently indicated to harbor important functions in several types of human malignancy [17, 28–30]. Recently, dysregulation of SNHG4 has been found in various human malignancies, such as prostate cancer, osteosarcoma, cervical cancer, and hepatocellular carcinoma [17–21]. For example, lncRNA SNHG4 promotes cervical tumorigenesis and progression by sponging miR-148a-3p and upregulating the expression of c-Met [18]. SNHG4 boosts osteosarcoma growth by competitively binding miR-224-3p [20]. Presently, we explored the expression pattern and function of SNHG4 in RCC cell lines and human tissue samples and found that SNHG4 expression pattern in RCC tissue samples was significantly associated with several clinicopathological features of RCC patients (aggressive T stage, node invasion, metastasis status, UISS, and poor tumor grade). Univariate and multivariate Cox regression analysis indicated that SNHG4 expression was an independent prognostic factor for poor survival and tumor recurrence in patients with RCC.

Previous studies have suggested that lncRNAs play essential roles in modulation of the malignant phenotypes of cancer cells. To explore the functions of SNHG4 in RCC cells, we performed the loss-of-function and gain-of-function experiments in two RCC cell lines (769-P and ACHN). Our results implicated that the downregulation of SNHG4 prohibited RCC cell proliferation, migration, and invasion, and enhanced cell apoptosis in vitro. We also showed knockdown of SNHG4 inhibits RCC cell tumorigenesis in vivo. On the contrary, overexpression of SNHG4 led to the contrary results both in vitro and in vivo. All these data potentially implicate SNHG4 acts as an oncogenic function in the pathogenesis and progression of RCC.

Mechanically, increasing evidence has revealed that lncRNA in the cytoplasm acts as a ceRNAs, which bind miRNAs to release such miRNAs targeted genes from degradation in human cancers [31]. In the previous studies, SNHG4, as a miR-377 sponge, was demonstrated to elevate the expression of ZIC5 in prostate carcinoma [19]. Furthermore, in osteosarcoma cell lines, the SNHG4/miR-224-3p/DOCK7 axis was implicated in the regulation of cell proliferation, migration, and invasion [20]. Here, we sought to explore whether SNHG4 may also serve as a ceRNA to regulate the carcinogenesis and advancement of RCC. Using the bioinformatics database (lnclocator and starBase 3.0), we found that SNHG4 was primarily located in the cytoplasm of RCC cell lines and contained theoretical binding sites of miR-204-5p. Further experiments confirmed that SNHG4 could directly bind to miR-204-5p and weaken the expression levels of miR-204-5p in RCC cell lines. Results from recent researchers have indicated that miR-204-5p harbors the tumor suppressive roles in several types of human cancer, including RCC [32–34]. In line with previous results, our data confirmed the downregulation of miR-204-5p in RCC tissue specimens and cell lines and the tumor‐suppressive role of miR-204-5p in RCC. Furthermore, correlation analysis confirmed an inverse association between miR-204-5p and the abundance of SNHG4 in RCC tissue samples. Importantly, RIP assay analysis demonstrated that SNHG4 and miR-204-5p were positively correlated in the Ago2‐containing RNA‐induced silencing complex (RISC). Based on these data, we concluded that SNHG4 could competitively interact with miR-204-5p and prohibit the expression of miR-204-5p in RCC cell lines.

We further explored the downstream targetting gene of miR-204-5p in RCC cell lines. The present data showed that RUNX2, a well-known transcription factor that participated in tumor EMT, migration, and invasion [35, 36], was recognized as the downstream target of miR-204-5p and had a competition with SNHG4 to interact with miR-204-5p in RCC cell lines. Furthermore, according to the correlation analysis, we found that RUNX2 mRNA level had an inverse association with miR-204-5p but a positive association with SNHG4 in RCC tissue specimens. Based on the luciferase reporter assay, we eventually verified RUNX2 as a direct target of miR-204-5p in RCC cell lines. Last but not least, rescue assays conducted in RCC cell line (ACHN) further certified that SNHG4 regulates the malignant biological phenotypes of RCC cell lines via the miR-204-5p/RUNX2 axis.

## Conclusion

Taken together, the present study reveals that lncRNA SNHG4 harbors an oncogenic function that promotes carcinogenesis through acting as ceRNA for miR‐205‐5p. Our data support the idea that SNHG4/miR-204-5p/RUNX2 axis plays an essential role in RCC progression and potentially work as a therapeutic target.

## Supplementary information

**Additional file 1**: Additional Tables and Figures.

## Data Availability

All data generated or analyzed during this study are included in this published article.

## Abbreviations

ccRCC: Clear cell renal cell carcinoma
lncRNA: Long noncoding RNA
snoRNA: Small nucleolar RNA
SNHG: Small nucleolar RNA host gene
FBS: Fetal bovine serum
siRNA: Small interfering RNA
LV: Lentivirus
UTR: Untranslated region
Mut: Mutant-type
RIP: RNA immunoprecipitation
CCK-8: Cell counting kit-8
IHC: Immunohistochemistry
TT: Tumor tissue
ANT: Adjacent nontumor tissue
TCGA: The cancer genome atlas
KIRC: Kidney renal clear cell carcinoma
RFS: Relapse-free survival
OS: Overall survival
HR: Hazard ratio
CI: Confidence interval
ceRNA: Competing endogenous RNA
RISC: RNA‐induced silencing complex
