# Supplementary material for: Long noncoding RNA SNHG4 promotes renal cell carcinoma tumorigenesis and invasion by acting as ceRNA to sponge miR-204-5p and upregulate RUNX2
Source: Cancer Cell Int. 2020 Oct 19;20:514. doi: 10.1186/s12935-020-01606-z (PMC7574175; doi:10.1186/s12935-020-01606-z)

Long Noncoding RNA SNHG4 Promotes Renal Cell Carcinoma Tumorigenesis and Invasion by Acting as ceRNA to Sponge miR-204-5p and Upregulate RUNX2

Jie Wu, Tingting Liu, Lulu Sun, Shaojin Zhang, Gang Dong

Table S1: Clinicopathologic characteristics of the renal cell carcinoma patients

| Parameters |  | % |
| --- | --- | --- |
| Age, years |  |  |
| <55 | 43 | 43.4 |
| ≥ 55 | 56 | 56.6 |
| Gender |  |  |
| Male | 69 | 69.7 |
| Female | 30 | 30.3 |
| Pathological T stage |  |  |
| pT1+ pT2 | 66 | 66.7 |
| pT3+ pT4 | 33 | 33.3 |
| Pathological N stage |  |  |
| pN0 | 72 | 72.7 |
| pN1 | 27 | 27.3 |
| Distant metastasis |  |  |
| No | 91 | 91.9 |
| Yes | 8 | 8.1 |
| TNM Stage** |  |  |
| I | 31 | 31.3 |
| II | 8 | 8.1 |
| III | 8 | 8.1 |
| IV | 3 | 3.0 |
| Grade |  |  |
| 1+ 2 | 77 | 77.8 |
| 3+4 | 22 | 22.2 |
| UISS category |  |  |
| low risk | 43 | 43.4 |
| mediate risk | 41 | 41.4 |
| high risk | 15 | 15.2 |

Table S2: The sequences of the primers included in this manuscript

|  | Forward premier (5’-3’) | Reverse premier (5’-3’) |
| --- | --- | --- |
| Primers for qRT-PCR | | |
| RUNX2 | TGGACGAGGCAAGAGTTT | CTTCTGGGTTCCCGAGGT |
| GAPDH | GGTGAAGGTCGGAGTCAACGG | GAGGTCAATGAAGGGGTCATTG |
| U6 | GCTTCGGCAGCACATATACT | GTGCAGGGTCCGAGGTATTC |
| U1 | GGGAGATACCATGATCACGAAGGT | CCACAAATTATGCAGTCGAGTTTCCC |
| SNHG4 | GCAGGTGACAGTCTGCATGT | TTTTAAGTCCCCTACCCCCATC |
| miR-204-5p | TTCCCTTTGTCATCCTATGCCT |  |

**Supple. Figure legends**

Fig. S1 The clinical significance of SNHG4 expression in human RCC tissue samples according to TCGA-KIRC dataset.

A, SNHG4 expression was significantly higher in tumor tissues than in normal renal tissues. B-D, High expression of SNHG4 was significantly associated with node invasion (B), poor tumor grade (C), tumor stage (D). E, qRT-PCR assay showed the expression levels of SNHG4 in five RCC cell lines and HK-2, a renal epithelial cell line. Data are presented as means ± standard deviation from triplicate experiments. A t-test was used to evaluate the statistical significance as compared to the control. *, *P* < 0.05; **P < 0.01; ***P < 0.001.


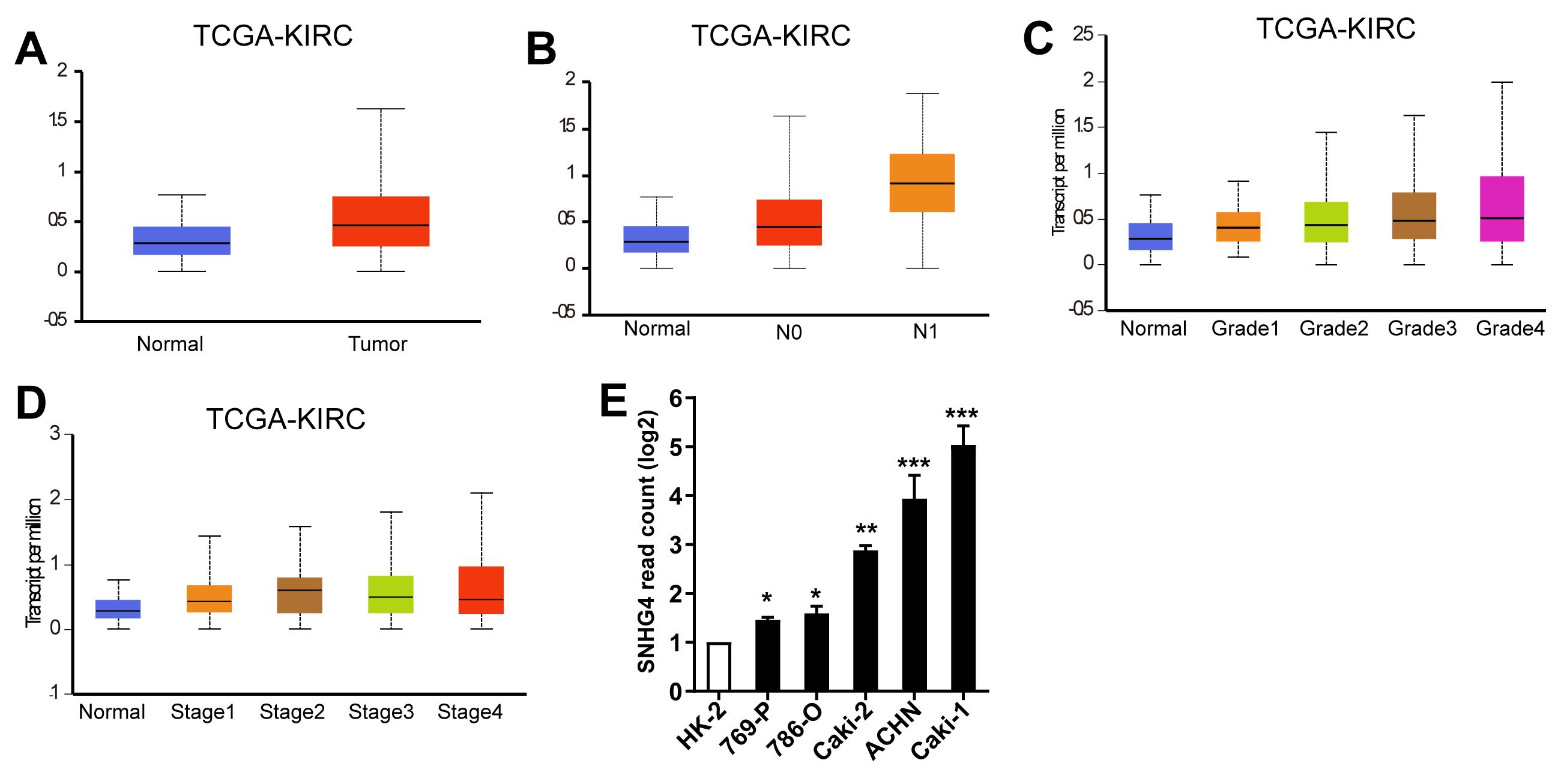


Fig. S2

A, Based on the Starbase 3.0 online software, the predicted 40 miRNAs which might bind to SNHG4 were shown. B, miR-204-5p expression possessed a negative correlation with SNHG4 in human RCC tissue samples according to TCGA-KIRC dataset. C, miR-204-5p expression was significantly decreased in tumor tissues compared with normal renal tissues according to TCGA-KIRC dataset. D, qRT-PCR assay analysis of the expression levels of miR-204-5p in five RCC cell lines and HK-2 cell line. E qRT-PCR assay analysis of the expression levels of miR-204-5p in 769-P and ACHN cell lines after transfection with the indicated miR vectors. Data are presented as means ± standard deviation from triplicate experiments. A t-test was used to evaluate the statistical significance as compared to the control. *, *P* < 0.05; **P < 0.01; ***P < 0.001.


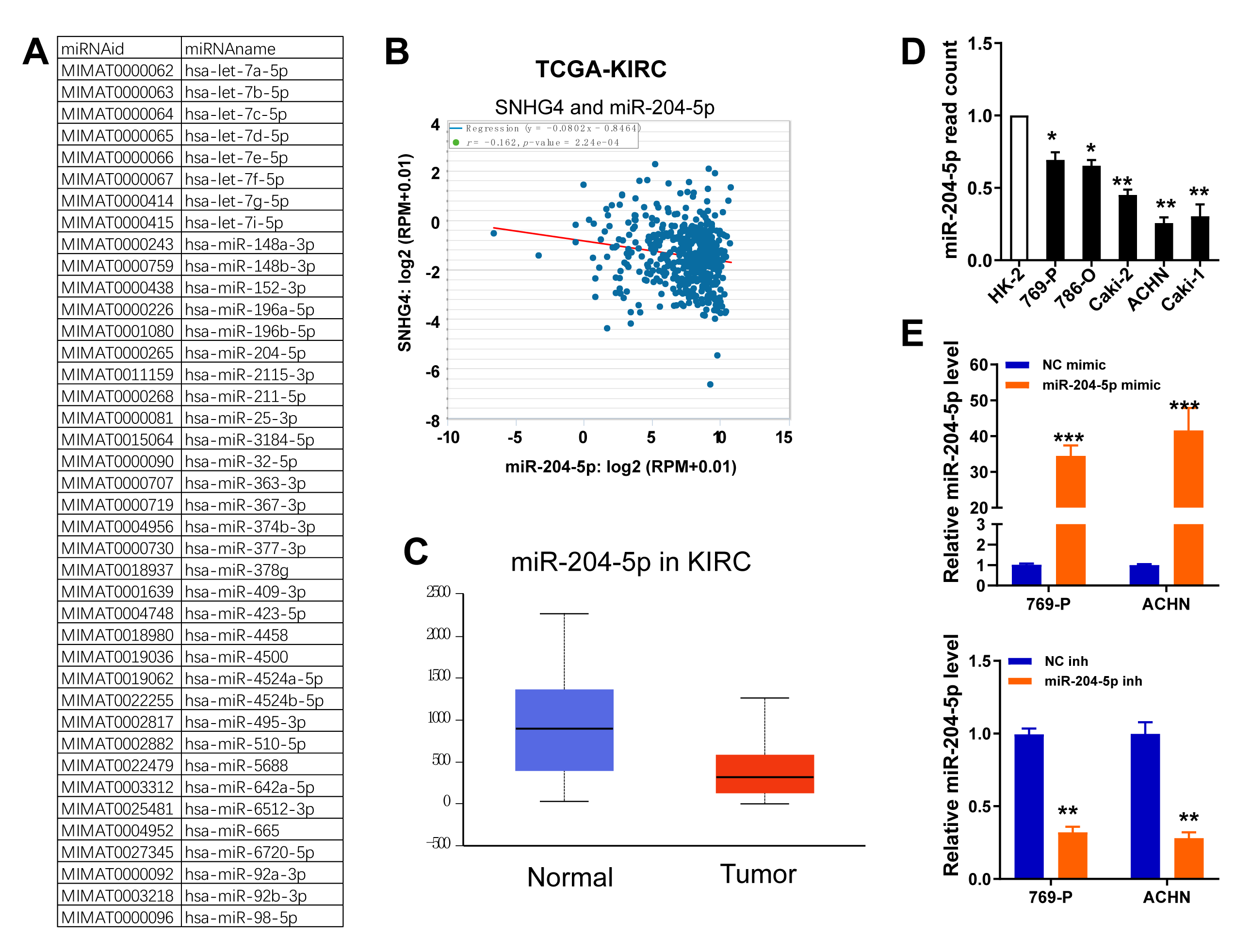


Fig. S3

A, RUNX2 expression was significantly increased in tumor tissues compared with normal renal tissues according to TCGA-KIRC dataset. B-E, High expression of RUNX2 was significantly associated with node involvement (B), poor tumor grade (C), advanced tumor stage (D), and poor overall survival (E), according to TCGA-KIRC dataset. F, qRT-PCR assay analysis of the expression levels of RUNX2 in five RCC cell lines and HK-2 cell line. Data are presented as means ± standard deviation from triplicate experiments. A t-test was used to evaluate the statistical significance as compared to the control. *, *P* < 0.05; **P < 0.01; ***P < 0.001.


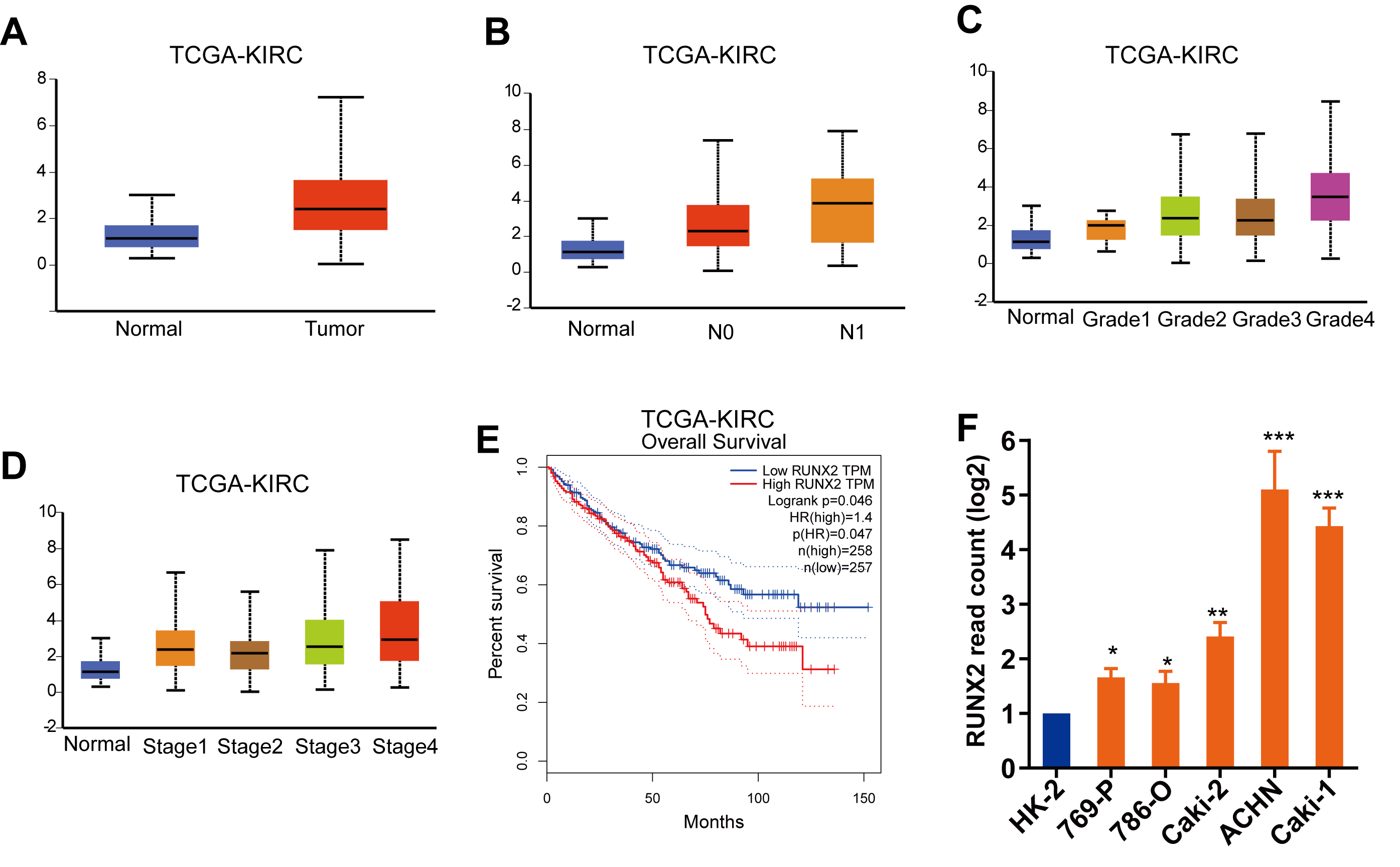

Supplement: Supplementary file 1 — Additional file 1: Additional Tables and Figures. [file 12935_2020_1606_MOESM1_ESM.doc]
